# Correlation of amplification and overexpression of the c-*myc* oncogene in high-grade breast cancer: FISH, *in situ* hybridisation and immunohistochemical analyses

**DOI:** 10.1038/sj.bjc.6601703

**Published:** 2004-03-30

**Authors:** J Blancato, B Singh, A Liu, D J Liao, R B Dickson

**Affiliations:** 1 Institute for Molecular and Human Genetics, 3970 Reservoir Road, NW, Washington DC 20007, USA; 2 Lombardi Comprehensive Cancer Center, 3970 Reservoir Road, NW, Washington DC 20007, USA; 3 Biometry and Mathematical Statistics Branch, National Institute of Child Health and Human Development, 9000 Rockville Pike, Bethesda, MD 20892-7510, USA; 4 Department of Radiation Oncology, Karmanos Cancer Institute, Wayne State University, Detroit, MI, USA; 5 Department of Oncology, Georgetown University Medical Center, 3970 Reservoir Road, NW, Washington DC 20007, USA

**Keywords:** c-*myc*, breast cancer, gene amplification, gene expression

## Abstract

In this study, we analysed gene amplification, RNA expression and protein expression of the c-*myc* gene on archival tissue specimens of high-grade human breast cancer, using fluorescent *in situ* hybridisation (FISH), nonradioactive *in situ* hybridisation and immunohistochemistry. The specific question that we addressed was whether expression of c-*Myc* mRNA and protein were correlated with its gene copy amplification, as determined by FISH. Although c-*Myc* is one of the most commonly amplified oncogenes in human breast cancer, few studies have utilised *in situ* approaches to directly analyse the gene copy amplification, RNA transcription and protein expression on human breast tumour tissue sections. We now report that by using the sensitive FISH technique, a high proportion (70%) of high-grade breast carcinoma were amplified for the c-*myc* gene, irrespective of status of the oestrogen receptor. However, the level of amplification was low, ranging between one and four copies of gene gains, and the majority (84%) of the cases with this gene amplification gained only one to two copies. Approximately 92% of the cases were positive for c-*myc* RNA transcription, and essentially all demonstrated c-myc protein expression. In fact, a wide range of expression levels were detected. Statistically significant correlations were identified among the gene amplification indices, the RNA expression scores and protein expression scores. c-*myc* gene amplification, as detected by FISH, was significantly associated with expression of its mRNA, as measured by the intensity of *in situ* hybridisation in invasive cells (*P*=0.0067), and by the percentage of invasive cells positive for mRNA expression (*P*=0.0006). c*-myc* gene amplification was also correlated with the percentage of tumour cells which expressed high levels of its protein, as detected by immunohistochemistry in invasive cells (*P*=0.0016). Thus, although multiple mechanisms are known to regulate normal and aberrent expression of c-*myc*, in this study, where *in situ* methodologies were used to evaluate high-grade human breast cancers, gene amplification of c-*myc* appears to play a key role in regulating expression of its mRNA and protein.

The c-*myc* oncogene has been shown to be amplified and/or overexpressed in many types of human cancer (Marcu *et al*, 1992; Nass and Dickson, 1997; Nesbit *et al*, 1999; Liao and Dickson, 2000). Numerous experiments *in vivo* have also causally linked aberrant expression of this gene to the development and progression of cancer in different body sites (Marcu *et al*, 1992; Nass and Dickson, 1997; Nesbit *et al*, 1999; Liao and Dickson, 2000). However, several critical issues regarding the significance of c-*myc* in human cancer still remain obscure. First, even for a given type of malignancy, the frequencies of the alterations of c-*myc* at the cytogenetic and expression levels vary greatly from one report to another (Liao and Dickson, 2000). For instance, the frequencies of its amplification, mRNA and protein overexpression in breast cancer vary between 1–94, 22–95 and roughly 50–100%, respectively, among different reports (Liao and Dickson, 2000). Thus, it is still unclear to what extent this gene is altered at the cytogenetic level and at different expression levels in breast carcinoma.

One controversial issue pertains to the prognostic value of c-*myc* gene alterations in cancer. The central role of c-Myc protein in accelerating cell proliferation, documented by many early studies, has led to a general concept for many types of cancer that amplification or overexpression of this gene may be associated with a more aggressive tumour and a poorer patient survival (Berns *et al*, 1992; Marcu *et al*, 1992; Sato *et al*, 1995; Nass and Dickson, 1997; Nesbit *et al*, 1999; Visca *et al*, 1999; Liao and Dickson, 2000). However, many reports have shown an opposite correlation (Sikora *et al*, 1985, 1987; Watson *et al*, 1986; Polacarz *et al*, 1989; Voravud *et al*, 1989; Williams *et al*, 1990; Melhem *et al*, 1992; Pietilainen *et al*, 1995; Diebold *et al*, 1996; Smith and Goh, 1996; Augenlich *et al*, 1997; Bieche *et al*, 1999), while other studies do not support either of these conclusions. For instance, gene amplification or overexpression of c-Myc protein has also been shown to associate with a better tumour differentiation or a better patient survival for cancer of the testis, ovary, bile ducts, colon and breast (Sikora *et al*, 1985, 1987; Watson *et al*, 1986; Polacarz *et al*, 1989; Voravud *et al*, 1989; Williams *et al*, 1990; Melhem *et al*, 1992; Pietilainen *et al*, 1995; Diebold *et al*, 1996; Smith and Goh, 1996; Augenlich *et al*, 1997; Bieche *et al*, 1999). This controversy does not appear to be related completely to the cancer type, since both positive (Berns *et al*, 1992; Visca *et al*, 1999) and negative (Williams *et al*, 1990; Melhem *et al*, 1992; Pietilainen *et al*, 1995; Smith and Goh, 1996; Augenlich *et al*, 1997; Bieche *et al*, 1999) correlations have been reported for colon cancer and breast cancer. More interestingly, c-Myc overexpression has been shown to predict a poorer prognosis for cutaneous melanoma, but a favourable outcome for uveal melanoma (Grover *et al*, 1997; Chana *et al*, 1998a, 1998b, 1999; Grover *et al*, 1999). These data indicate different roles of c-Myc, even in the same type of tumour, perhaps depending upon different tissue microenvironments.

Another controversial issue concerns the nuclear–cytoplasmic localisation of c-Myc. Studies of neoplasms of the colon, testis, ovary and liver have shown that predominantly nuclear localisation of c-Myc tends to occur in benign lesions, while cytoplasmic localisation tends to occur in more malignant tumours (Sikora *et al*, 1985; Sundaresan *et al*, 1987; Melhem *et al*, 1992; Sasano *et al*, 1992; Yuen *et al*, 2001). Whether these patterns of subcellular localisation of c-Myc tend to reflect the malignant status of breast cancer remains an enigma.

A recent study of the impact of DNA amplification on gene expression patterns in breast cancer used mRNA and DNA from 14 breast cancer cell lines. Analysis was conducted with a 13 000 cDNA clone array for gene expression measurement and a Comparative Genomic Hybridisation (CGH) microarray for gene copy number measurements. This study also included known breast cancer genes, such as c*-myc*, *HER/2-neu* and *aib1* (Hyman *et al*, 2002). Interestingly, 44% of the most highly amplified genes were also overexpressed at the mRNA level. Consistent with this pattern, c-*Myc* gene copy number and its expression levels showed a statistically significant (*α*=0.020) correlation in this microarray study of breast cancer cell lines. Another study, by Pollack and colleagues, used microarray analysis and BAC array CGH of RNA and DNA (respectively) extracted from intermediate grade human breast tissues, and tested for amplification and expression of c-Myc (among other genes). This study demonstrated that two out of 37 specimens were both amplified and overexpressed, while others were either amplified or overexpressed, but not both. The authors of this study suggested that contaminating stromal tissue may compress the fluorescence ratios leading to underestimates of gene amplification and overexpression (Pollack *et al*, 2002).

To more clearly address the importance of gene amplification and expression of c-Myc in human breast cancer, we used *in situ* methodologies, which can clearly distinguish stromal and carcinoma components. We studied the amplification and over expression of the c-*myc* gene with fluorescent *in situ* hybridisation (FISH), non-radioactive *in situ* hybridisation (ISH) and immunohistochemical (IHC) approaches on paraffin-embedded biopsy sections of untreated, high-grade breast cancer. It was observed that 70, 92 and 70% of the cancer cases exhibited c-*myc* gene amplification, its mRNA overexpression and its protein over expression, respectively. In most of the cases (84%) that showed gene amplification, the c-*myc* gene gained only one to two copies, which is consistent with c-*myc* FISH data from other studies. Unlike some oncogenes, such as N-*myc,* which typically demonstrates gene amplification copy numbers of greater then 10 in neuroblastoma, and *HER-2/neu* (Sartelet *et al*, 2002), whose copy numbers range up to 14–40 in breast carcinomas (Isola *et al*, 1999), gene copy numbers of c-*myc* are not as greatly increased. In the study noted earlier, using breast cancer cell line CGH array and cDNA microarray expression analysis, it was demonstrated that the most dramatically increased expression levels were associated with large gene copy number increases, although low-level gains and losses had a significant influence on gene expression dysregulation (Hyman *et al*, 2002). Only one study has been published (Pollack *et al*, 2002) that has begun to determine if these findings are directly relevant to actual human breast tumour tissues, since many of the genetic changes in tissue culture cell lines are more extreme than those displayed in primary tumour material. Furthermore, the relationships among gene amplification, mRNA expression and c-Myc protein expression were not explored in prior human breast cancer cell line and tumour tissue studies (Hyman *et al*, 2002; Pollack *et al*, 2002).

In our human breast tumour tissue study, a high correlation was found between c-*myc* FISH and ISH, for both percentage of staining (*P*<0.0067) and intensity positive cells (*P*<0.0006). In addition, c-*myc* gene copy amplification by FISH was correlated with c-Myc protein expression positive cells by IHC (*P*<0.0016). These results support the idea that c-Myc overexpression of both mRNA and protein is related to the copy number of the *c-myc* DNA amplification. We show in this study that amplification and overexpression of c-Myc occur with high frequency in high-grade human breast cancer tissues.

## MATERIALS AND METHODS

### Materials

Formalin-fixed, paraffin-embedded tissue blocks of breast carcinoma and normal breast tissue were obtained from the Histopathology and Tissue Shared Resource at the Lombardi Comprehensive Cancer Center (LCCC), at Georgetown University Medical Center. The criteria for tumour selection were the following: negative progesterone receptor status, metastases to auxiliary lymph nodes and high grade (Elston Score >7). The oestrogen receptor status of the tumours was known from archived pathology reports. The parameters were chosen from our prior meta-analysis (Deming *et al*, 2000), as indications of a high likelihood of c-*myc* gene amplification. Normal breast tissue specimens were from reduction mammoplasty. Serial sections (5 *μ*m) for FISH, ISH and IHC were prepared by the LCCC Histopathology and Tissue Shared Resource.

### FISH

A dual-label FISH technique was used (Jenkins *et al*, 1997). Slides were baked overnight at 60°C to assure adherence of the sample. Tissue sections were deparaffinised with two successive, 10 min xylene washes, and then dehydrated in a graded ethanol series of 70, 80 and 95% at room temperature. Samples were then digested with 4% pepsin (Sigma, St Louis, MO, USA) at 45°C for 10 min. DNA probes used were an alpha satellite probe to chromosome 8, labelled with biotin, and a *c-myc* probe, labelled with digoxigenin (Ventana, Tucson, AZ, USA). Codenaturation was performed at 90° for 10 min on a hot plate. Hybridisation was at 37°C for 12–16 h. Detection of signals was accomplished with an antiavidin antibody labelled with Texas Red, and an antidigoxigenin antibody conjugated to fluorescein (Ventana, Tucson, AZ, USA). Slides were postwashed in 2 × SSC at 72°C for 5 min and counterstained with DAPI to visualise cell nuclei. Results were viewed and quantified with a Zeiss Axiophot fluorescence microscope, equipped with appropriate filters and an Applied Imaging Cytovision system (Pittsburgh, PA, USA). In this approach, the c*-myc* unique sequence probe was visualised as a green signal and the control probe for the chromosome 8 centromere was red, thus easily being distinguished when scored.

One serial section from each tumour sample was stained with haematoxylin and eosin and first reviewed by a pathologist (BS), to help identify the tumour area of the section. This procedure ensured that the tumour cells, but not the normal cells, were counted. Nuclei of up to 50 tumour cells were scored from each FISH-stained section, independently by two investigators. Hybridisation signals were averaged, and the amplification index was presented as the number of c-*myc* signals divided by the number of chromosome 8 centromere signals. A 1.8-fold increase was used as the criterion to judge the presence of c-*myc* gene amplification.

### *In situ* hybridisation

*In situ* hybridisation (ISH) was carried out with a nonradioactive method, described previously (Liao *et al*, 2000a, 2000b). One serial section from each specimen was hybridised overnight at 60°C with riboprobes, that were *in vitro* transcribed from the antisense or sense strand of an approximately 300 bp cDNA of human c-*myc* (ATCC, Manassas, VA, USA), labelled with digoxigenin-conjugated UTP. The sections were then incubated with an antibody against digoxigenin, followed by incubation with a second antibody conjugated to alkaline phosphatase. The signal was visualised by colour development with 5 bromo-4-chloro-3-indolyl phosphate and nitroblue tetrazolium. All reagents were purchased from Boehringer Mannheim, Indianapolis, IA. To control the signal specificity, two serial sections were mounted on the same slide for hybridisation with the antisense and sense probes, respectively. ISH was given an intensity and percentage scores, based on intensity of positive staining and number of cells staining, respectively. Intensity scores were assigned 0, 1, 2 and 3, and percentage scores were assigned as 1- 1–25, 2- 26–50, 3- 51–75 and 4- 76–100%.

### Immunohistochemistry

Immunohistochemical staining (IHC) was performed using an avidin–biotin complex (ABC) method described previously (Liao *et al*, 1998). One serial section of each specimen was deparaffinised and blocked with 3% peroxide. Antigens were retrieved by heating slides in a microwave oven in 50 mM citrate buffer, pH 6.4, at boiling temperature, for 12 min. After blocking with 6% normal goat serum, the section was incubated with a mouse monoclonal antibody to human c-Myc (9E10, Sigma Chemical Company, St Louis, MO, USA) at 1 : 100 dilution for 2 h, followed by 1 h incubation with a second antibody conjugated with biotin (Vector Laboratories Inc., Burlingame, CA, USA). The section was then incubated with peroxidase-conjugated avidin (Dako, Corporation, Carpinteria, CA, USA) for 30 min, followed by colour development with diaminobenzidine and peroxide. All procedures were carried out at room temperature. To control the signal specificity, serial sections from 10 tumour samples were also stained using an alternate c-Myc antibody (C19 from Santa Cruz Biotechnology Inc., Santa Cruz, CA, USA) at 1 : 60 dilution. This antibody resulted in focally positive staining in the tumour, but the staining intensity was weaker. To control the signal specificity, serial sections were made from five selected positive cases which were subjected to the same staining procedure, with a normal mouse IgG to replace the c-Myc antibody. This control staining did not give rise to a signal, demonstrating the specificity of the c-Myc antibody signal. IHC staining was given an intensity and percentage score based upon the intensity of positive staining and number of cells staining. Intensity scores were assigned 0, 1, 2 and 3 and percentage scores were assigned as 1- 1–25, 2- 26–50, 3- 51–75 and 4- 76–100%. Determinations were made of cellular localisation of c-Myc antibody staining to cytoplasm and/or nucleus in normal and invasive cells within each breast tumour specimen.

### Statistical analyses

For each analysis of gene copy amplification (FISH), mRNA expression (ISH) and protein expression (IHC), all cases were first grouped as positive or negative to calculate the percentages of positive cases and negative cases, as described (Zar, 1974). Fisher's exact test was used to compare percentages, and two-sample *t*-test or Wilcoxon rank test was used to compare average scores. Both ISH and IHC were given intensity and percentage scores, based on intensity of positive staining and number of cells staining, respectively. As noted earlier, intensity scores were assigned 0, 1, 2 and 3 and percentage scores were assigned as 1- 1–25, 2- 26–50, 3- 51–75 and 4- 76–100%. A score of >2 for either intensity of staining or percentage of cells positive by ISH was assigned as high. For IHC, an intensity score of >1 was assigned as high and a percentage score of >3 was categorised as high. Each amplification index was paired with its corresponding mRNA expression score to calculate the coefficient *r*. The same method was used to estimate the association of the amplification indices with the c-Myc protein expression levels, and the association of the mRNA expression levels with the protein expression levels. A *P*-value of 0.05 or less was used to determine the statistical significance in all analyses. In all, 54 pairs of normal *vs* invasive tissues were analysed using McNemars χ^2^ test to determine if there was a difference in cellular localisation of c-Myc antibody signal to nuclear or cytoplasmic compartments.

## RESULTS

### FISH analysis of gene amplification

**Figure 1:**
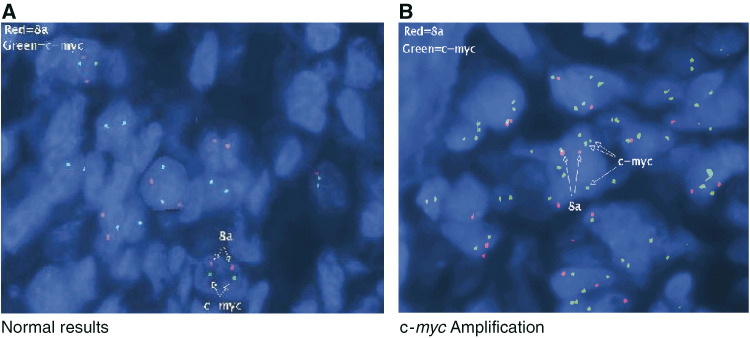
FISH analysis of c-*myc* amplification in tumour cells from breast tumour tissue sections. FISH probe for human c-*myc* unique-sequence is seen as green, while the normal control signal, a centromeric probe signal for chromosome 8 is shown in red. The nuclei of tumour cells were visualised by DAPI counter-staining. (**A**) 1 : 1 copy ratio of c-*myc* to chromosome 8 (c-*myc*/8 centromere), indicating no amplification of c-*myc* in tumour cells. (**B**) 1 : 3 copy ratio of c-*myc* to chromosome 8 (c-*myc*/8 centromere), a moderate amplification of the c-*myc* gene.

Amplification of the c-*myc* gene was measured by a FISH test in 46 cases of breast cancer; Figure 1

demonstrates cells with no amplification (one copy of c-*myc* /one copy of chromosome 8 centromere, and a moderate amplification a 3/1 ratio). Amplification was calculated by the number of *c-myc* signals divided by the number of chromosome 8 alpha satellite signals. A 1.8-fold increase cut-off was used to judge gene amplification. As shown in Table 1

**Table 1:** c-*myc* gene copy amplification analysis by FISH in poor prognosis human breast tumour samples

**Amplification index (#*c-myc* signals/# control signals)+**	**Percentage of samples with FISH ratios in each category**	
1.0–1.7	30%	14 out of 46
1.8–1.99	20%	Nine out of 46
2.0–2.9	39%	18 out of 46
>3.0	11%	Five out of 46

Analysis was conducted on 46 individual paraffin-embedded tissue samples with negative progesterone receptor status, positive lymph node involvement and high tumour grade.+Normal control ratio is 1.

, 32 out of 46 (70%) cases were gene amplified for c-*myc*, whereas only 30% (14/46) of the cases showed amplification indices lower than the cut-off value. The amplification indices for most (84%, or 27/32) cases with gene amplification, ranged between 1.8- and three-fold, indicating that the locus gained up to two copies of c-*myc* in the majority of the cases. The percentage of cases with gene gains of three copies or higher was 11% (five out of 46) of total cases analysed, or near 16% (five out of 32) of the cases with gene amplification, including one case (2% of total cases or 3% of the cases with gene amplification) with the highest index of 5 (a gain of four copies).

In all, 28 of the breast carcinomas in this study were ER negative, and 14 were ER positive. The average c-*myc* gene amplification score was 1.896 (s.e.=0.196) for ER positive and 2.201 (s.e.=0.157) for ER negative. Although ER-negative tumours had a slightly higher average c-*myc* score, the difference was not statistically significant (two-sided *P*=0.252 from two-sample *t*-test and 0.251 from Wilcoxon rank test), consistent with the results of our prior meta-analysis of the literature (Deming *et al*, 2000).

### *In situ* hybridisation analysis of c-*myc* mRNA expression

A total of 51 breast cancer samples were studied for c-Myc mRNA expression, with non radioactive *in situ* hybridisation (ISH). ISH results were assigned intensity and percentage scores based upon signal intensity of positive staining and number of cells staining within the sample, respectively. As shown in Table 2

**Table 2:** c-myc mRNA *in situ* hybridisation (ISH) results

**Staining intensity**	**0**	**1**	**2**	**3**	**Percent positivity**	**1**	**2**	**3**	**4**
Number of tumour samples in each category *N*=51	1	6	25	19	Number of tumour samples in each level category *N*=51	1	3	5	42
									

**Figure 2:**
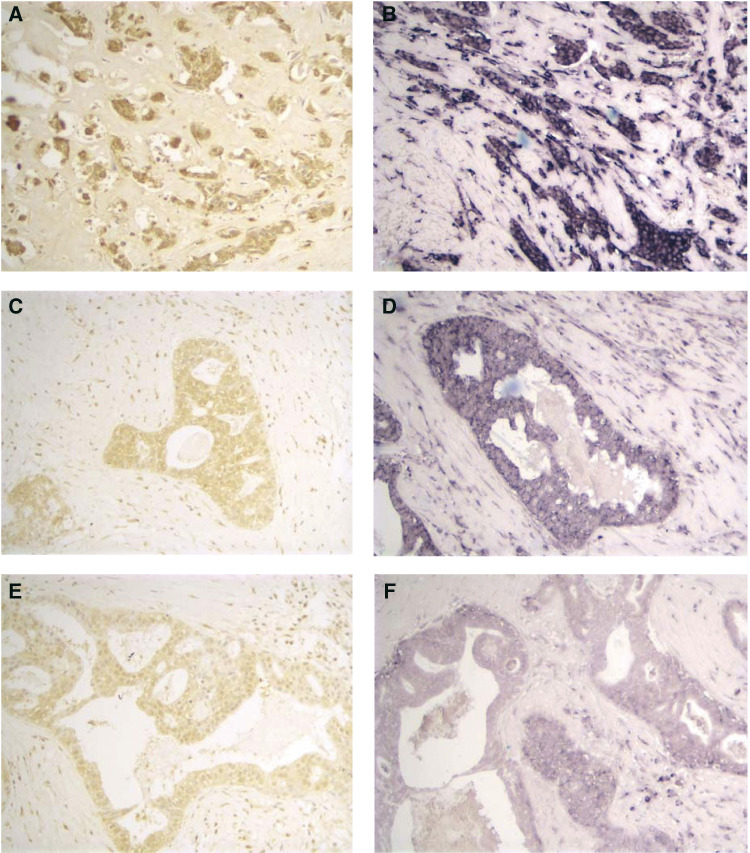
Immunohistochemical staining and *in situ* hybridisation for c-Myc of three sets of invasive ductal carcinoma. (**A**, **C** and **E**) High (3+), intermediate (2+) and low (1+) level of staining by immunohistochemistry for c-Myc. (**B**, **D** and **F)** High (3+), intermediate (2+) and low (1+) level of staining by *in situ* hybridisation.

In all, 51 human high-grade breast carcinomas were analysed to determine the relationships between c-Myc mRNA expression and *c-myc* gene *in situ* hybridisation results. Data are shown in two ways in the above table. First, overall staining intensity of c-Myc-positive cells was scored as 0, 1, 2, 3 (low to high), and the number of tumour samples at each level of staining indicated on the line below. Next, the percentage of tumour cells staining was scored as 0, 1, 2, 3, 4 (low to high %, as discussed in Materials and Methods). The number of tumours at each level of percent cell positivity for c-Myc is then indicated on the line below.

, 86% (44 out of 51) tumours were scored as high in intensity, and 92% (47out of 51) had more than 51% positive cells, also considered as highly increased c-Myc expression. mRNA expression was heterogeneous in the breast tumour tissue, and no morphologic subtype was predominant in the high or low categories. One case showed no c-Myc ISH staining. In 79% (38/48) of cases, epithelia in normal mammary glands adjacent to the tumour also showed a high intensity of staining. In three cases, no staining was seen in the normal terminal duct lobular units. Figure 2

shows representative fields of high, medium and low *c*-*myc* mRNA expression levels in invasive ductal carcinoma samples.

### Association of FISH and ISH

c-Myc scores were dichotomised as binary variables (high or low), and a score of 2 or higher was categorised as high on ISH. A score higher than median was categorised as high from FISH studies. These dichotomised scores are depicted in Table 3

**Table 3:** Correlations between c-*myc* gene copy number (FISH) mRNA expression (ISH)

	**FISH**	
	**Low**	**High**
(A) ISH (% cells)		
Low	*1*	3
High	*19*	*18*
		*P*=0.0067
		
(B) ISH (intensity)		
Low	*2*	*5*
High	*18*	*16*
		*P*=0.0006

Serial sections of high-grade human breast carcinomas were scored for c*-myc* gene copy number (FISH, Table 1) and mRNA expression (ISH, Table 2). In (A), a positive correlation (*P*=0.0067) was observed between tumour samples with a high percentage of cells demonstrating mRNA expression and a high c*-myc* gene copy number. A score of 2 or higher was classified as high on ISH, and a score of median or greater was categorised as high on FISH. In (B), a positive correlation (*P*=0.0006) was shown between a high level of intensity for c-Myc RNA expression a high and c*-myc* gene copy number. Note that a pairwise comparison of FISH and ISH was not possible for all cases, due to incomplete overlap of cases analysed with each assay.

. A Fisher's exact test was performed for comparing binary responses to see if there was any association between FISH and ISH. It was found that the FISH score was significantly associated with percentage of staining in the invasive cells (*P*=0.0067, two-sided McNemar's test) and also with the intensity score on ISH (*P*=0.0006, two-sided).

### Immunohistochemical staining of c-Myc proteins

In total, 51 breast carcinomas, which were subjected to FISH analysis, and all of which also had been analysed for c-*myc* mRNA by *in situ* hybridisation, were also analysed for the expression of c-Myc protein, using immunohistochemical staining with the 9E10 antibody. IHC results were assigned an intensity and percentage score based on intensity of positive staining and number of cells staining, respectively. Intensity scores were assigned 0, 1, 2 and 3 and percentage scores were assigned as 0, 1- 0–25, 2- 26–50, 3- 51–75 and 4- 76–100. For IHC, an intensity score of >1 was assigned as high and a percentage score of >3 was categorised as high. Figure 2 shows examples of high, medium and low levels of c-myc antibody staining in invasive ductal carcinoma samples. In 34 cases, normal tissue was seen; 30 of these showed cytoplasmic staining and 22 had nuclear staining in terminal ductal lobular units. In all, 12 cases showed 1+, 14 cases 2+ and four cases 3+ cytoplasmic staining. *In situ* hybridisation revealed positive staining in 46 out of 49 cases with normal tissue. Seven cases showed 1+, 13 cases showed 2+ and 26 cases showed 3+ staining by ISH. Both immunohistochemistry and *in situ* hybridisation showed diffuse positivity in adipocytes.

Table 4

**Table 4:** c-Myc immunohistochemistry (IHC) results

**Staining intensity**	**0**	**1**	**2**	**3**	**Percent positivity**	**1**	**2**	**3**	**4**
Number of tumour samples in each category	15	13	20	3	Number of tumour samples in each category	2	2	1	29
									

In all, 51 high-grade human breast carcinomas were analysed to determine the relationships between c-Myc protein expression and c*-myc* gene *in situ* hybridisation results. Data are shown in two ways in the above table. First, overall staining intensity of c-Myc-positive cells was scored as 0, 1, 2, 3 (low to high), and the number of tumour samples at each level of staining is indicated on the line below. Next, in a random subset of these cases, the percentage of tumour cells staining was scored as 0, 1, 2, 3, 4 (low to high %, as discussed in Materials and methods). The number of tumours at each level of percent cell positivity for c-Myc is indicated on the line below.

shows the staining pattern for the cohort. In all, 70% (36 out of 51) of cases showed high intensity of staining for c-Myc protein, while 85% (29 out of 34) of cases with detectable staining had more than 76% positive cells, also considered as high expression. To verify the staining specificity, serial sections from 10 tumour specimens that were positive for 9E10 antibody were also stained using the C19 rabbit polyclonal anti-c-Myc antibody. Results revealed a staining pattern similar to 9E10. However, the staining intensity with C19 was weaker than 9E10. The specificity of these two antibodies was verified by Western blots in previous studies (Persons *et al*, 1997; Liao *et al*, 2000b). Figure 2 shows results of c-Myc *in situ* hybridisation and immunohistochemistry studies on samples considered to demonstrate low, moderate and high levels of c-Myc expression. Analysis of c-Myc protein localisation results in the nucleus or cytoplasmic compartments of normal and invasive cells within the tumours revealed that nuclear staining was positive in 41% of normal cells, compared to 22% of invasive cells (statistical significance at *P*=0.01 by McNemar's two-sided χ^2^ test). The increase in relative cytoplasmic localisation of c-Myc protein, comparing normal (53.7%), to invasive cells (61.1%) was not significantly different. Thus, the data are consistent with partial exclusion of c-Myc from the nuclei of invasive breast cancer cells.

The FISH score was significantly associated with the percentage positivity of invasive cells, as seen on IHC studies of c-Myc. However, 40% of tumours displayed a low index of c-*myc* gene amplification, but still expressed high levels of c-Myc protein (Table 6

**Table 6:** Correlation between c-Myc protein expression (IHC) and c*-myc* gene copy number (FISH)

	**FISH**	
IHC (% cells)	**Low**	**High**
		
Low	3	0
High	10	15
		*P*=0.0016

Consecutive serial sections of high-grade human breast tumours were scored for c-*myc* gene copy number or protein expression, by immunohistochemistry (IHC). IHC scores were defined in the Materials and methods section. Data were analysed for correlations between the results. A highly significant correlation was observed between high c-Myc protein expression (IHC) between percent cells positive and high c*-myc* gene amplification (FISH). *P*=0.0016 from two-sided McNemar's test. Note that for 15 cases, no staining for c-Myc could be detected; these negative cases were not included in the correlation presented, above.

), indicating the possibility of other mechanisms of over expression unrelated to gene amplification in at least some tumours. The FISH score was not significantly associated with the intensity of IHC staining in the invasive cells (not shown), in contrast to the IHC percentage positivity score.

## DISCUSSION

Although there have been many reports on c-*myc* amplification in human breast cancer (Liao and Dickson, 2000), there are only two published studies involving application of the FISH technique to unfixed, frozen sections (Persons *et al*, 1997; Visscher *et al*, 1997), and one prior study using FISH on an archival human tissue microarray (Schraml *et al*, 1999). Another recent study applied FISH to evaluate c-*myc* amplification in ductal carcinoma *in situ* (DCIS) (Aulmann *et al*, 2002). Using the FISH technique on formalin-fixed, paraffin-embedded sections, we now show that 70% of high-grade breast cancer samples bear c-*myc* gene copy amplifications. Interestingly, the above-mentioned study, using FISH and focusing on DCIS, detected amplification of c-*myc* in only 20% of cases, but found a correlation of c-*myc* with increased tumour size and proliferation (Aulmann *et al*, 2002).

The level of amplification of c-*myc* in our study ranged between one and four additional copies of the gene; the majority (84%) of the cases with the gene amplification gained only one to two copies, also consistent with FISH data reported for c-*myc* copy amplification in human metastatic prostate carcinoma tissues (Jenkins *et al*, 1997). The relationship between the level of c-*myc* gene copy amplification and the level its increased mRNA expression has been examined previously in breast cancer cell lines (Hyman *et al*, 2002). In general, it has been concluded that the two scores coordinate for c-*myc*, as is the case for many breast cancer genes. However, only 44% of the highly amplified genes, in general, showed increased RNA expression, and only 10.5% of the highly overexpressed genes were gene copy-amplified in the cell line study (Hyman *et al*, 2002). Another analysis was conducted to study of relationships between gene amplification and expression of 6095 genes in 37 intermediate grade human breast tumours. This study demonstrated that 62% of the highly amplified genes also showed elevated expression; overall, a two-fold change in DNA copy number was associated with a 1.5-fold change in mRNA levels. Overall, 12% of the variation in gene expression in the breast tumours studied was associated with gene copy number variation (Pollack *et al*, 2002). Further study of additional human breast tumours, at precisely defined grades and stages, will be necessary in order to more fully define the relationships between DNA copy numbers and expression of genes. The studies we report here indicate higher levels of c-Myc gene amplification and expression, than other previous reports in breast cancer. We believe that this is probably the result of our analysis of individual tumour cells in a well-defined set of high-grade breast tumours. Prior c-Myc expression and amplification microarray studies used tumour specimens which contain normal stromal components, potentially underestimating amplification and expression levels of the invasive tumour components (Pollack *et al*, 2002).

Our study reports a percentage of tumours gene amplified for c-*myc* (using FISH in high-grade tumours) that is much higher than the average figure (15.5%) reported in the literature (Isola *et al*, 2002). Most of the prior studies have employed the relatively insensitive Southern blot technique, and were reviewed in a recent meta-analysis (Deming *et al*, 2000). Consistent with this prior literature background, a recent study of 94 lobular and ductal breast cancers assessed amplification of c-*myc* by using a semiquantitative PCR assay and protein expression, with densitometry, after Western blot. These data showed c-*myc* gene amplification in 21% of tumours (Jenkins *et al*, 1997), using assays not based on *in situ* discrimination of tumour *vs* nontumour cells. The lower frequency of c-*myc* in this prior study is in contrast with the data we present here, and could be the result of the higher sensitivity and precision of the FISH and immunohistochemical methods, as distinct from quantitative PCR and Western blot densitometry. In addition, the 70% of amplified tumours in our study is also much higher than the 12% reported by Schraml *et al* (1999), using a c-*myc* FISH test on a tissue microarray. This large difference may be because the arrays are prepared from cores of paraffin-embedded tissue, as small as 0.6 mm in diameter which may contain too few tumour cells for complete analysis of amplification of a gene, such as c-*myc*. c-*myc* is known to be quite heterogeneous in its gene amplification within individual tumours (in contrast to *HER2/neu*, for example) (Persons *et al*, 1997).

Most previous reports on the expression of c-*myc* mRNA have utilised Northern blot, dot blot or PCR-based approaches, while just a few involved *in situ* hybridisation, which were primarily performed on frozen tissue sections (Liao and Dickson, 2000). Normal breast tissue is dominated by adipose cells, differing greatly from tumour tissue in its epithelial cellularity. Thus, normal and tumour tissues may not be rigorously compared by techniques involving RNA extraction from total tissue. Therefore, conclusions such as ‘increased expression’ may be more difficult to make from studies with Northern blot, dot blot and PCR-based techniques that require RNA extraction from tissues that have not been fastidiously micro-dissected for selection of tumour cells. Using a more sensitive, nonradioactive *in situ* hybridisation (ISH) approach on formalin-fixed, paraffin-embedded sections, we report herein high expression of c-*myc* mRNA in 92% of high-grade breast carcinomas. This figure is much higher than the recently reported data (22%), obtained by using a real-time RT–PCR method (Bieche *et al*, 1999). Dilution of the RNA from epithelium by the RNA from adipose in normal breast tissue in this latest prior report may be one of the possible explanations for this large difference.

In conclusion, the present study shows that approximately 70, 92 and 70% of biopsies of untreated high-grade breast cancer exhibit c-*myc* gene amplification, mRNA overexpression and protein overexpression, respectively. In most cases (84%), with gene copy amplification, the c-*myc* gene gains one to two additional copies. c-*myc* gene amplification was significantly associated with expression of its mRNA (both by intensity in invasive cells and by percentage positivity in invasive cells), and with expression of its protein (by percentage positivity in invasive cells). However, our data were also consistent with the prior literature on c-Myc (reviewed in Nass and Dickson, 1997; Liao and Dickson, 2000), indicating complex transcriptional, post transcriptional, translational and post-translational control of c-Myc expression *in vitro*. Specifically, in Table 5

**Table 5:** Nuclear/cytoplasmic localisation of c-Myc comparing normal and invasive cells

**Normal cells (frequency percent)**	**Invasive cells (frequency percent)**		**Total**
(A) Nuclear localisation			
	−	+	
−	28	4	32
+	14	8	22
Total	42	12	54
			
(B) Cytoplasmic localisation			

−	12	13	25
+	9	20	21
Total	21	33	54

In all, 54 pairs (normal *vs* invasive) of tissues were analysed to answer the questions of (1) whether positivity of nuclear cells in normal tissues is different from that in invasive cells, and similarly (2) whether positivity of cytoplasmic cells in normal tissues is different from that in invasive cells. The data are summarised in the above contingency tables. In all, 22 normal cell specimens were positive for c-Myc staining (40.71%), compared to 12 specimens (22.2%) in invasive cells. The difference is statistically significant (*P*=0.01) by McNemar's χ^2^ test (two-sided).

we observed that in 40% of the high-grade tumours tested, c-Myc protein was expressed at high levels, despite a lack of its gene amplification.

It will be interesting to analyse lower grade tumours and premalignant lesions, with the same measurement tools, to determine if this c-*myc* amplification pattern is different, comparing different steps in onset and progression of the disease. Specifically, prior studies in fibroblasts and in human mammary epithelial cells (Liao *et al*, 1998, 2000a, 2000b) have demonstrated that only a subtle deregulation of expression of c-Myc is sufficient to allow genomic instability. These prior cell biologic findings raise the question of whether c-Myc protein expression precedes or follows its gene amplification during the course of the natural history of breast cancer. It will also be interesting for future studies of lower grade breast cancers and premalignant lesions to determine whether there is evidence of nuclear exclusion of c-Myc protein. Indeed, nuclear exclusion of c-Myc in high-grade tumours could serve to attenuate its functions in later stages of disease progression (Liao and Dickson, 2000).
